# What is the neurobiology of schizophrenia?

**DOI:** 10.1017/S1092852924000518

**Published:** 2024-10-30

**Authors:** Michael A. Cummings, Ai-Li W. Arias, Stephen M. Stahl

**Affiliations:** 1 University of California, Irvine, CA, USA; 2 University of California, Riverside, CA, USA; 3 University of California, San Diego, CA, USA; 4 University of Cambridge, Cambridge, UK

**Keywords:** Neurobiology, schizophrenia, developmental dementia, dementia praecox, brain changes, dopamine, glutamate

## Abstract

Schizophrenia spectrum disorders are brain diseases that are developmental dementias (dementia praecox). Their pathology begins in utero with psychosis most commonly becoming evident in adolescence and early adulthood. It is estimated they afflict the U.S. population at a prevalence rate of approximately 0.8%. Genetic studies indicate that these brain diseases are about 80% determined by genes and about 20% determined by environmental risk factors. Inheritance is polygenic with some 270 gene loci having been identified as contributing to the risk for schizophrenia. Interestingly, many of the identified gene loci and gene polymorphisms are involved in brain formation and maturation. The identified genetic and epigenetic risks give rise to a brain in which neuroblasts migrate abnormally, assume abnormal locations and orientations, and are vulnerable to excessive neuronal and synaptic loss, resulting in overt psychotic illness. The illness trajectory of schizophrenia then is one of loss of brain mass related to the number of active psychotic exacerbations and the duration of untreated illness. In this context, molecules such as dopamine, glutamate, and serotonin play critical roles with respect to positive, negative, and cognitive domains of illness. Acutely, antipsychotics ameliorate active psychotic illness, especially positive signs and symptoms. The long-term effects of antipsychotic medications have been debated; however, the bulk of imaging data suggest that antipsychotics slow but do not reverse the illness trajectory of schizophrenia. Long-acting injectable antipsychotics (LAI) appear superior in this regard. Clozapine remains the “gold standard” in managing treatment-resistant schizophrenia.

Schizophrenia spectrum disorders are a cluster of psychotic brain diseases that afflict approximately 0.4% to nearly 2.0% of persons in various worldwide populations.^1^ ^,^^2^ In 2019, the direct and indirect annual costs of schizophrenia were estimated at $343.2 billion in the United States alone.^3^ Moreover, in addition to a substantial economic burden on society as a whole, the schizophrenia spectrum disorders impose a variety of devastating personal and familial burdens, including but not limited to social isolation, disruption of education, unemployment, homelessness, intrafamilial violence, entanglement in the legal system, incarceration, increased injury and illness, and a shortened life span.^4^ ^,^^5^ Given the costly and disastrous effects of the schizophrenia spectrum disorders, Emil Kraepelin, who first characterized these psychotic disorders, described them as dementia praecox or early dementia.^6^ In the remainder of this review, we will consider the neurobiology underlying a cluster of brain diseases that can be conceptualized under an umbrella as a group of developmental dementias with similar core pathologies but heterogeneous variations in clinical detail.

The human genome was first published in 2001.^7^ Since then, researchers have been working to identify protein-coding genes. The number of such genes is presently estimated at between 19,000 and 20,000.^8^ Within the human genome, some 270 gene loci have been associated with schizophrenia spectrum disorders, with 108 risk genes being identified as single nucleotide polymorphisms.^9^ The most obvious genetic associations have been with genetic variations in the major histocompatibility complex. Besides polymorphisms, structural variants in the form of copy number variants, such as microdeletions and microduplications have a very high impact in a subset of patients. These variations are mainly microdeletions on 1q21.1, 2p16.3, 3q29, 15q13.3, and 16p11.2, as well as a large deletion on 22q11.21 and a microduplication on 16p11.2.^10^ Importantly, many of the genes and gene loci implicated in schizophrenia are involved in areas such as cell differentiation, cell regulation, cell maturation, cell migration, orientation of cells, the structure of cell receptors, cell adhesion, and, in the case of neurons, development of neural networks.^11^ ^,^^12^ Additionally, those gene foci that are part of the histocompatibility complex play critical roles in immune identity and control of inflammatory processes.^8^ ^,^^13^ ^,^^14^

Although schizophrenia spectrum disorders are heavily genetically determined, it is thought that about 20% of the risk for overt illness is determined by environmental factors such as maternal stress during pregnancy, in utero infection exposure, childhood illnesses, childhood adversity, and childhood or adolescent exposure to drugs such as methamphetamine or cannabis.^1^ ^,^^15^ Many of these environmental risk factors may influence the occurrence and phenotypic development of schizophrenia via epigenetic processes, such as gene promotion or inhibition of other genes using small peptides or short ribonucleic acid (RNA) sequences, methylation of deoxyribonucleic acid (DNA), or modulation of the acetylation of histone (protein involved in the winding and unwinding of DNA strands for copying).^16^ ^,^^17^ Moreover, while no gene therapies currently exist for schizophrenia spectrum disorders, interventions in selected environmental risk factors hold promise for altering the phenotypic presentation of schizophrenia, as well as risk of overt illness in both present and future generations.^18^ ^,^^19^

The human central nervous system begins as a simple tube formed from neural crest cells. This relatively simple structure, however, then undergoes a complex and elegant series of steps to become the brain and spinal cord.^20^ The brain is formed by overfolding of the cephalad portion of the neural tube with glial cells laying down the structural form of the brain and providing trails of chemical markers for motile neuroblasts to follow to their cortical and subcortical positions.^21^ During the second trimester of pregnancy, neuroblasts (immature motile forms of later neurons) undergo rapid mitosis deep in the forming brain near the lateral ventricles. These neuroblasts then crawl to their later positions following neurotrophic markers and organize themselves into orderly neural assemblies.^20^ They then form neural networks by sprouting axons and dendrites. Initially, the number of connections is 2 to 5 times greater than the connections present in the mature brain. That is, exposure to the environment and the process of learning selects those pathways that will be reinforced and those that will be allowed to atrophy as the brain matures.^22^ ^-^^24^ The primary visual cortex is the first to mature at about 1 year of age, while the last areas to mature are the frontal and temporal lobes at between 18 the 25 years of age. Thus, the roughly 100 billion neurons of the central nervous system, along with their associated astrocytes, oligodendrogliocytes, and microglia, as well as other cell types, become the adult brain and spinal cord.^21^ ^,^^24^ ^,^^25^

In contrast, brain development and maturation in schizophrenia spectrum disorders is clearly abnormal. To begin, many of the neuroblasts produced during the second trimester of pregnancy fail to reach their correct positions, instead being found in post-mortem studies isolated deep within the white matter of the brain.^26^ ^,^^27^ Then, across childhood and adolescence individuals in the premorbid phase of schizophrenia exhibit excessive loss of neurons and synaptic connections, such that by the onset of overt psychosis some one-third to one-half exhibit clear atrophic changes and enlargement of the lateral ventricles on brain imaging.^28^ ^,^^29^ Ventricular enlargement, reflecting loss of brain tissue in schizophrenia, is illustrated below (Figure 1).

**Figure 1.**
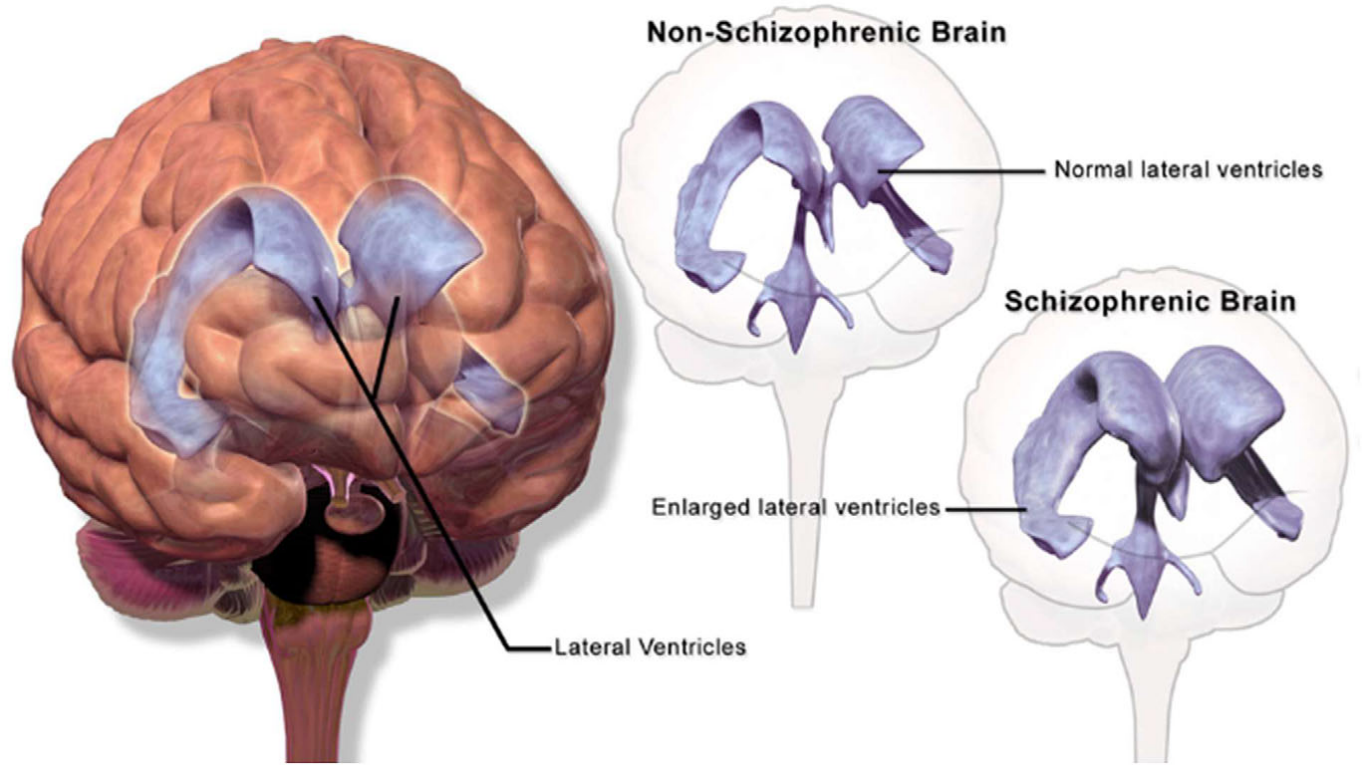
Ventricular enlargement/brain atrophy. openbooks.lib.msu.edu (open access).

Following the onset of overt illness, loss of brain mass continues and appears to be correlated with the duration of untreated illness and the number of psychotic exacerbations.^1^ ^,^^29^ ^,^^30^ At least a portion of the brain tissue loss associated with psychotic exacerbations or longer durations of untreated active psychosis appears to be mediated by inflammatory processes, including activation of microglia and invasion of the brain by macrophages.^14^ ^,^^31^ ^,^^32^ Interestingly, treatment of high-risk children (i.e., having 2 parents with schizophrenia spectrum disorders) with low-dose antipsychotic medications may reduce the rate of conversion to overt illness in adolescence.^33^ Nevertheless, it should be noted that some subsequent studies have failed to find evidence that antipsychotic treatment during the premorbid phase of schizophrenia is protective with respect to later development of overt schizophrenia.^34^ Better established appear to be observations that consistent antipsychotic treatment (eg, with LAIs) slows but does not reverse the deterioration of the brain in schizophrenia spectrum disorders.^35^ ^-^^37^

Clinically, the signs and symptoms of schizophrenia have been divided into positive, negative, and cognitive deficit domains.^1^ ^,^^38^ Positive signs and symptoms include hallucinations/illusions, delusional ideation, illogical thoughts and behavior, hyperactivity/agitation, and thought disorder.^1^ ^,^^38^ Negative signs and symptoms include apathy, lethargy, abulia, avolition, and social withdrawal.^39^ Cognitive deficits in schizophrenia spectrum disorders include deficits in attention, concentration, memory organization and recall, language processing, and executive functions such as self-awareness and social judgment.^38^ ^,^ ^40^ In addition to the developmental abnormalities and atrophic brain changes described earlier in this article, 2 neuromodulatory molecules, that is, dopamine and serotonin, appear to play important functional roles in schizophrenia spectrum disorders.^41^ ^,^^42^ Below, we will consider 3 neural networks with respect to the positive, negative, and cognitive domains of schizophrenia.

Positive signs and symptoms appear to arise in part from excessive dopamine stimulation of mesostriatal projections to temporal lobe association cortices and related structures (formerly termed the mesolimbic pathway).^41^ ^,^^43^ This excessive stimulation of limbic D_2_ dopamine receptors, in turn, appears to arise from a failure of inhibition by gamma aminobutyric (GABA) interneurons in the frontal cortex. And failure of M_4_ acetylcholine receptors on the cell bodies of the relevant mesostriatal dopamine neurons.^41^ ^,^^44^ This is illustrated as follows (Figure 2).

**Figure 2.**
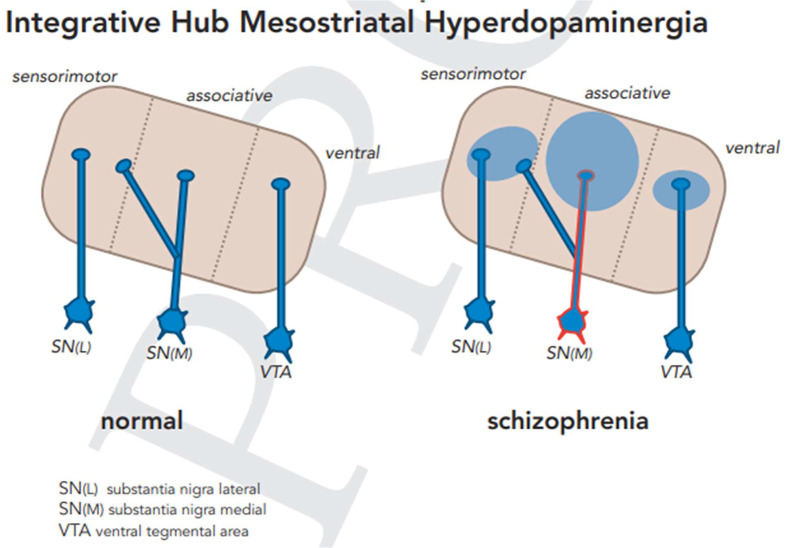
Mesostriatal dopaminergic hyperactivity. Stahl, S. Stahl’s Essential Psychopharmacology, 5^th^ Edition, Chapter 4, p. 93.

Excessive serotonin (5-hydroxytryptamine) stimulation of 5HT_2A_ receptors may add to positive psychotic signs and symptoms, especially visual hallucinations, in schizophrenia.^41^ ^,^^45^ This is illustrated as follows (Figure 3).

**Figure 3.**
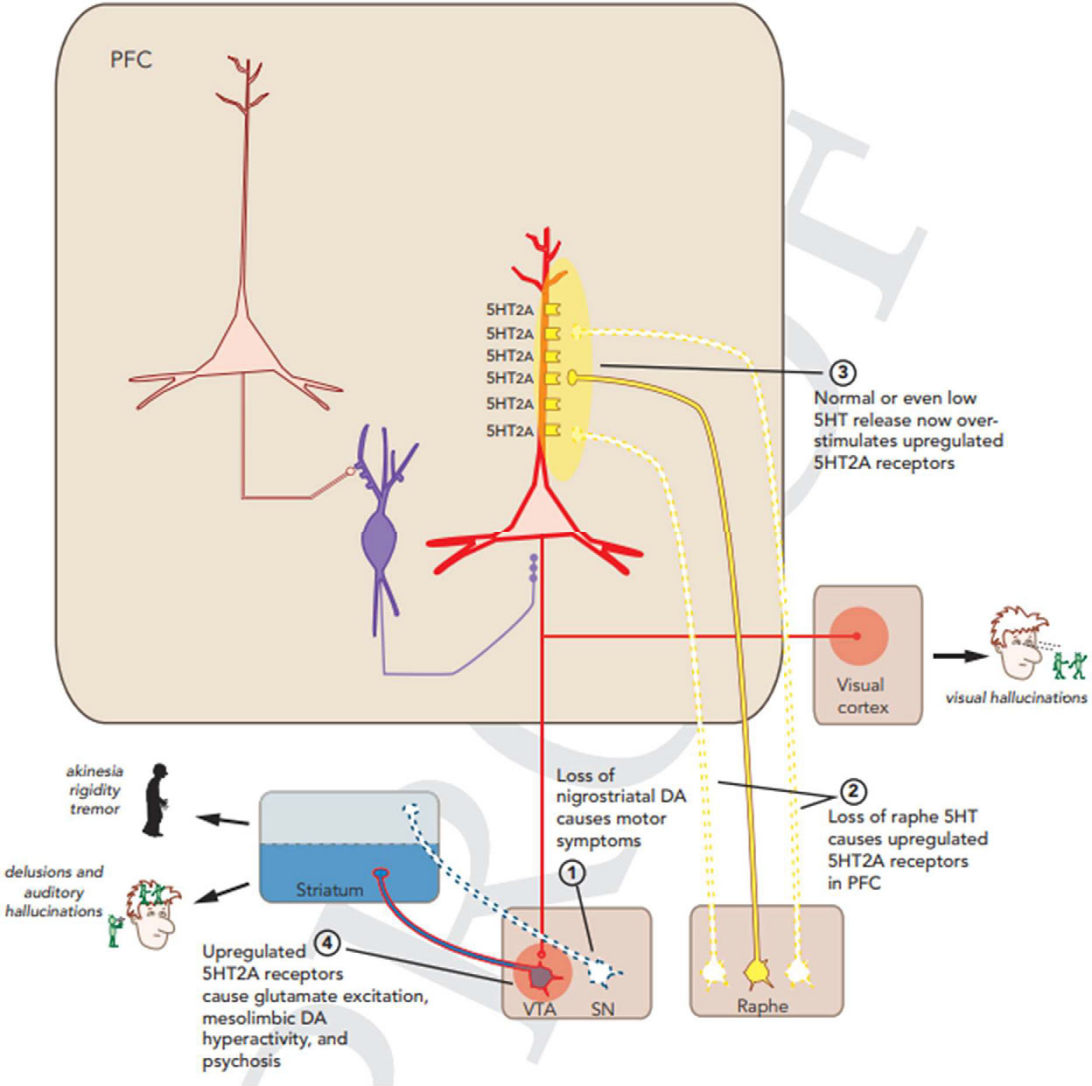
5HT_2A_ serotonergic hyperactivity. Stahl, S. Stahl’s Essential Psychopharmacology, 5^th^ Edition, Chapter 4, p. 136.

Finally, it appears that in addition to previously described developmental pathologies and atrophic changes, inadequate stimulation of frontal lobe D_1_ and D_3_ dopamine receptors contributes to the negative symptoms and cognitive impairments of schizophrenia spectrum disorders, including anosognosia (unawareness of illness).^41^ ^,^^46^ This is illustrated as follows (Figure 4).

**Figure 4.**
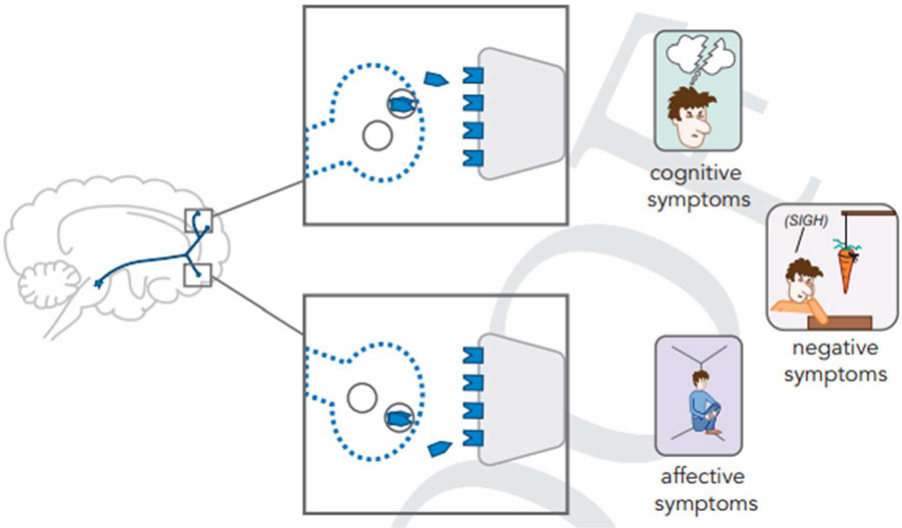
Mesocortical dopaminergic hypoactivity. Stahl, S. Stahl’s Essential Psychopharmacology, 5^th^ Edition, Chapter 4, p. 95.

Importantly, all antipsychotic medications appear capable of ameliorating psychotic symptoms, with the largest effects being on positive signs and symptoms.^47^ In particular, LAIs appear superior in preventing relapse and, thereby, illness progression, morbidity, and mortality.^35^ ^,^^48^ In the near future, a new class of antipsychotics likely starting with xanomeline/trospium may be able to presynaptically modulate dopamine release in mesostriatal projections by targeting the M_4_ acetylcholine auto-receptor.^44^ Among the antipsychotics, clozapine remains the “gold standard” of treatment in several areas, that is, management of treatment-resistant illness, reduction of violence, reduction of suicide risk, and enhancement of cognitive executive functions.^49^ ^,^^50^ Clozapine also appears to be unique in that it likely acts by exerting effects upstream of the mesostriatal dopamine neurons by improving glutamate signal transduction.^51^ ^,^^52^

Summary: Schizophrenia spectrum disorders are a group of related psychotic developmental dementias (dementia praecox) characterized by positive, negative, and cognitive signs and symptoms usually beginning in adolescence or early adulthood. Illness is mediated by a combination of developmental and atrophic changes in brain structure and defects in the signal transductions of glutamate, gamma amino butyric acid (GABA), acetylcholine, dopamine, and serotonin. Importantly, defects in neurotransmitter signal transduction provide targets for pharmacotherapy with antipsychotic medications. Critically, failure to provide consistent antipsychotic treatment early in the course of illness (eg, with LAIs) promotes atrophic brain pathology and deterioration of the illness course. Finally, while all antipsychotic medications can ameliorate acute signs and symptoms, clozapine shows superior efficacy in treating the positive, negative, and cognitive signs and symptoms of the schizophrenia spectrum disorders, as well as treatment resistance, violence, and suicide.
